# Inactivated rabies vaccines: Standardization of an *in vitro* assay for residual viable virus detection

**DOI:** 10.1371/journal.pntd.0008142

**Published:** 2020-03-25

**Authors:** Beatriz Lourenço Correia Moreira, Ana Paula Lappas Gimenez, Jorge Minor Fernandes Inagaki, Sonia Mara Raboni

**Affiliations:** 1 Center of Development and Production of Immunobiologicals, Instituto de Tecnologia do Parana (TECPAR), Curitiba, Parana, Brazil; 2 Postgraduate Program in Microbiology, Parasitology and Pathology, Universidade Federal do Parana, Curitiba, Parana, Brazil; 3 Virology Laboratory, Hospital de Clínicas, Universidade Federal do Parana, Curitiba, Parana, Brazil; Naval Medical Research Center, UNITED STATES

## Abstract

Human rabies, a neglected viral zoonosis, is preventable through domestic animals vaccination and post-exposure prophylaxis using inactivated rabies vaccines. During vaccine production, several mandatory *in vivo* quality control trials, such as potency, live virus, and safety, are responsible for the use of large numbers of laboratory animals. Over the years, global organizations encouraged the development of alternative methods to reduce, replace and refine the use of animals in the pharmaceutical industry. In this study we standardized an *in vitro* assay for determination of residual live virus combining viral isolation techniques with direct immunofluorescence detection and viral quantification by a molecular method. Standardization of viral recovery steps and quantification by RT-qPCR were performed and the combined method was shown to be 3 fold more sensitive than the *in vivo* assay. It was possible to identify viral suspensions cultures, which still had residual viable rabies virus particles, evidencing the importance to implement this method in quality control schemes of rabies vaccine production. In addition, this developed assay is more practical, inexpensive and less time consuming, producing results in just 4 days, which may allow greater agility in the internal quality control of the vaccine. The *in vitro* method may reduce 2/3^rd^ of laboratory animals numbers used for this purpose, since it can be applied in the intermediate quality control of inactivated rabies vaccine production.

## Author summary

Rabies is a viral disease that causes thousands of deaths worldwide every year, but can be preventable through vaccination of animals and humans. In the production of inactivated rabies vaccines, quality control assays use large amounts of laboratory animals. In order to reduce the number of animals in this practice, *in vitro* methods for residual live virus detection need to be developed. This study shows that combining two simple and effective techniques may be a safer and more accurate method for the detection of residual rabies virus than *in vivo* assays to evaluate inactivated vaccines and can be used as an intermediate quality control assay of vaccine production.

## Introduction

Rabies is a viral disease caused by the rabies virus (RABV), which belongs to the *Rhabdoviridae* family [1]. Rabies is responsible for approximately 60,000 deaths annually and yet it remains a neglected zoonosis that is endemic worldwide, except Antarctica and other isolated islands. African and Asian regions account for 95% of human rabies cases. In these regions, infected dogs whose saliva contains RABV are the main source of transmission through bites and scratches [2]. In other locations where urban rabies is under control, wildlife like bats, raccoons, foxes, and skunks are listed as the lead transmitters of rabies [3,4].

Although rabies is considered a deadly disease, characterized as severe encephalitis, it is preventable through extensive vaccination of dogs and cats and pre/post-prophylaxis treatments in exposed humans along with public health education [5]. Nowadays, vaccines used for human and veterinary rabies prevention are mostly inactivated vaccines, contrary to the wildlife oral vaccines that are produced with attenuated viruses [6]. To ensure the quality and consistency of inactivated rabies vaccine production, several international and local guidelines and monographs have specified various assays mandatory for the release of these vaccines. Among them, the potency, inactivation, safety and pyrogenicity tests are responsible for the use of a large number of laboratory animals [7].

Over the years, global organizations, such as the World Health Organization (WHO), the Office International des Epizooties (OIE), the European Center for the Validation of Alternative Methods (ECVAM) have encouraged the development of alternative methods to the use of animals in the pharmaceutical industry following the 3R principle (reduce, replace and refine) [7]. Among the assays required for batch release, the potency test being responsible for the majority of animals used, has received greater focus.

However, the residual live virus assay receives less attention, although it is critical for the safety assessment of vaccine batches. Therefore, the aim of this study was to standardize an *in vitro* immunofluorescence assay combined with molecular determination for viable residual virus detection in veterinary inactivated rabies vaccines during the intermediate steps of manufacture.

## Materials and methods

### Ethics statement

We conducted an experimental study to compare the findings of the *in vitro* and *in vivo* tests. This study was approved by the Ethical Commission on Animal Use of TECPAR, under No. 008/17, and addenda No. 006/18 and 001/19.

### Samples

The inactivated viral suspension (IVS) samples, used in this study were produced by Instituto de Tecnologia do Parana (TECPAR, Curitiba, Brazil). The samples were the intermediate products in the production of inactivated rabies vaccine. Rabies Pasteur Virus (PV, Pasteur Institute, FR) was propagated in Baby Hamster Kidney cells (BHK-21), viral suspension was collected, sucrose was added as stabilizer at a final concentration of 5% (v/v) (Merck, US) and the inactivation was performed with 0.02 volumes of beta-propiolactone (Natalex, PL) at 2–8°C with continuous shaking for 48 hours. Samples were stored at –80°C until further use. One finished vaccine dose contains no less than 1.0 x 10^3.50^ TCID_50_/mL of rabies virus and a potency of at least 1.0 IU/mL of rabies antigen.

Samples from other intermediate products such as formulated inactivated viral suspension (FIVS) with 0.01% thimerosal (Gihon, AR) and homogenized formulated inactivated viral suspension (HFIVS) with 0.02% aluminum hydroxide (Omega, BR) were also collected and stored at –80°C until use.

### Cell strain and culture

BHK-21 cells provided by Pasteur Institute, (FR) were cultured in Dulbecco’s Modified Eagle’s medium (D-MEM, Sigma-Aldrich, US) and HAM F-12 medium (Sigma-Aldrich, US) supplemented with 0.055% sodium pyruvate (Sigma-Aldrich, US), 2.44% sodium bicarbonate (Merck, US), 1.5% D-(+)-glucose (Sigma-Aldrich, US), 0.05% gentamicin sulfate (Inlab, BR) and 5% fetal bovine serum (FBS) (Laborclin, BR). Cells were transferred twice per week in T75 flasks at a density of 1.5 x 10^5^ cells/cm^2^ and incubated in a humidified incubator at 37°C with 5.0% CO_2_, unless stated otherwise.

### Virus

Rabies Pasteur Virus (PV, Pasteur Institute, FR) was used also as the working stock virus (WRV). The virus was prepared in BHK-21 cells cultured in T75 flasks for 3 days until a confluent monolayer was obtained, then inoculated with PV virus and incubated at 37°C for 5 days. Viral suspension was collected and stored at –80°C until further use. Virus titers were determined by titration in BHK-21 cells as previously described [8,9]. The virus titer of the working stock was 1.0 x 10^6^ TCID_50_/mL.

### Animals

Adult (21 days, 11–14 grams) and neonatal (4 to 6 days) Swiss-Webster mice were obtained from Instituto de Tecnologia do Parana (TECPAR, Curitiba, Brazil). For each test, 4 groups of ten adult mice and 2 groups of eight neonatal mice with their untreated mother were placed per cage. Mice were observed daily and were euthanized promptly as soon as classical rabies symptoms were confirmed (shaky movements, trembling, convulsions, paresis or paralysis, moribund state). All animal procedures were conducted according to recommendations of CONCEA–National Council for Control of Animal Experimentation (Brazil).

### Direct fluorescent antibody test (DFA)

Cells were cultured in 96-well plates for 24 h and then inoculated with samples diluted in culture medium for 2 h. Sample media were replaced by fresh medium and cells were incubated for 48–92 h. After that, the medium was completely removed, and cells were fixed with cold acetone 80% in ice bath for 15 min and the plates were allowed to dry in a safety cabinet for 10 min. Cells were then stained with fluorescein isothiocyanate (FITC)-labeled anti-rabies antibody (BioRad, FR) diluted 1/20 in 1/40.000 Evans Blue solution in PBS (Sigma-Aldrich, US) for 30 min at 37°C. Plates were washed twice with PBS and cells were examined using a fluorescence microscope IMT-2 (Olympus, JP). All wells were fully screened at 40x magnification and cells containing small fluorescent cytoplasmic granules were identified as antigen-positive after confirmation at 200x magnification. Positive and negative controls for DFA were done in each plate, where cells were incubated with WRV or simply media for controls in 2 wells.

### RT-qPCR assay

The molecular quantification of rabies virus was performed as described by Moreira et al, 2019. Briefly, 200 μL of samples at 0 h and 72 h of incubation was collected. Subsequently, viral RNA was isolated using PureLink Viral RNA/DNA Mini Kit (Invitrogen, US) following the manufacturer’s instructions in a final volume of 50 μL RNase free water and stored at –80°C until further testing. The duplex assay for the quantification of rabies virus targeting the viral nucleoprotein gene and BHK-21 β-actin as an internal control was performed as previously described by Moreira et al, 2019 [10]. All RT-qPCR assays were conducted on ViiA 7 Real-Time PCR System (AB Applied Biosystems, US).

### *In vivo* residual live virus assay

The *in vivo* residual live virus assay was conducted according to the Normative Instruction of the Ministry of Agriculture, Livestock and Supply (MAPA, BR) No. 228 of October 31^th^, 1988. Adult (21 days) and neonatal (4–6 days) mice were injected intra-cerebrally with pure and 1/10 diluted samples, with 30 and 10 μL, respectively, and monitored for 21 days for clinical symptoms and death. Animals deaths within 5 days of inoculation were considered unrelated to rabies, and therefor not included in the analysis. Mice showing clinical symptoms of rabies such as paralysis, convulsions and death by day 21 were characterized as rabies-related, and were included in the analysis [11].

### Confirmatory DFA for rabies diagnosis

DFA of brain samples for confirmatory rabies diagnosis, which is the gold-standard assay used in Brazil, was conducted according to the Normative Instruction of the Ministry of Agriculture, Livestock and Supply (MAPA, BR) No. 8 of April 12^th^, 2012. The brain samples were cut and pressed against a microscopy slide, fixed in acetone at –20°C for 15 min and dried at 20–25°C. Samples were then stained with fluorescein isothiocyanate (FITC)-labeled anti-rabies antibody (Pasteur Institute, São Paulo-BR) for 30 min at 37°C. Slides were washed with PBS, dried and prepared with 50% glycerol in PBS, for better conservation, and examined using a fluorescence microscope IMT-2 (Olympus, JP). Materials were examined at 40x magnification and samples containing small fluorescent cytoplasmic granules were identified as antigen-positive, and hence positive for rabies disease. Negative samples at 40x magnification were further analyzed at 200x to confirm the result. This procedure was performed in Center for Diagnosis “Marcos Enrietti”, ADAPAR, in Curitiba, Brazil, the reference public health laboratory for diagnosis of veterinary diseases.

### Transmission electronic microscopy of cell culture and brain

BHK-21 cells cultured in T25 flasks for 24 h at 37°C were inoculated with culture media, WRV, IVS samples that in the DFA test presented negative results (IVS–) or IVS samples that presented positive results (IVS+) for 2 h at 37°C, then incubated with fresh culture medium for 72 h at 37°C. Medium was removed, cells were trypsinized, washed with PBS, and fixed for 1 h with 4% paraformaldehyde and 2.5% glutaraldehyde in 0.1 M sodium cacodylate buffer and stored at 2–8°C until further processing. Brain samples of mice from the *in vivo* residual live virus assay were collected and cut into approximately 1x1 mm pieces, which were then fixed and stored as described above. Cell and brain samples were processed by the Cellular Biology Laboratory of the Carlos Chagas Institute of the Oswaldo Cruz Foundation in Curitiba, Brazil, following a procedure adapted from Haddad et al., 2007 [12]. Analysis was performed by Confocal and Electron Microscopy Platform–RPPI 017C –using a Jeol JEM1400-Plus transmission electron microscope (Jeol, JP).

### Histology

Formalin-fixed paraffin-embedded (FF-PE) brain samples were stained using a conventional haematoxylin-eosin (H&E) staining technique [13] and analyzed by photomicrographs acquired using a Zeiss Axio Scan.Z1 scanner (Carl Zeiss, US). This procedure was performed in the Experimental Pathology Laboratory of Universidade Pontifícia Catolica in Curitiba, Brazil.

## Results

### Determination of optimal culture conditions

**Fig 1:**
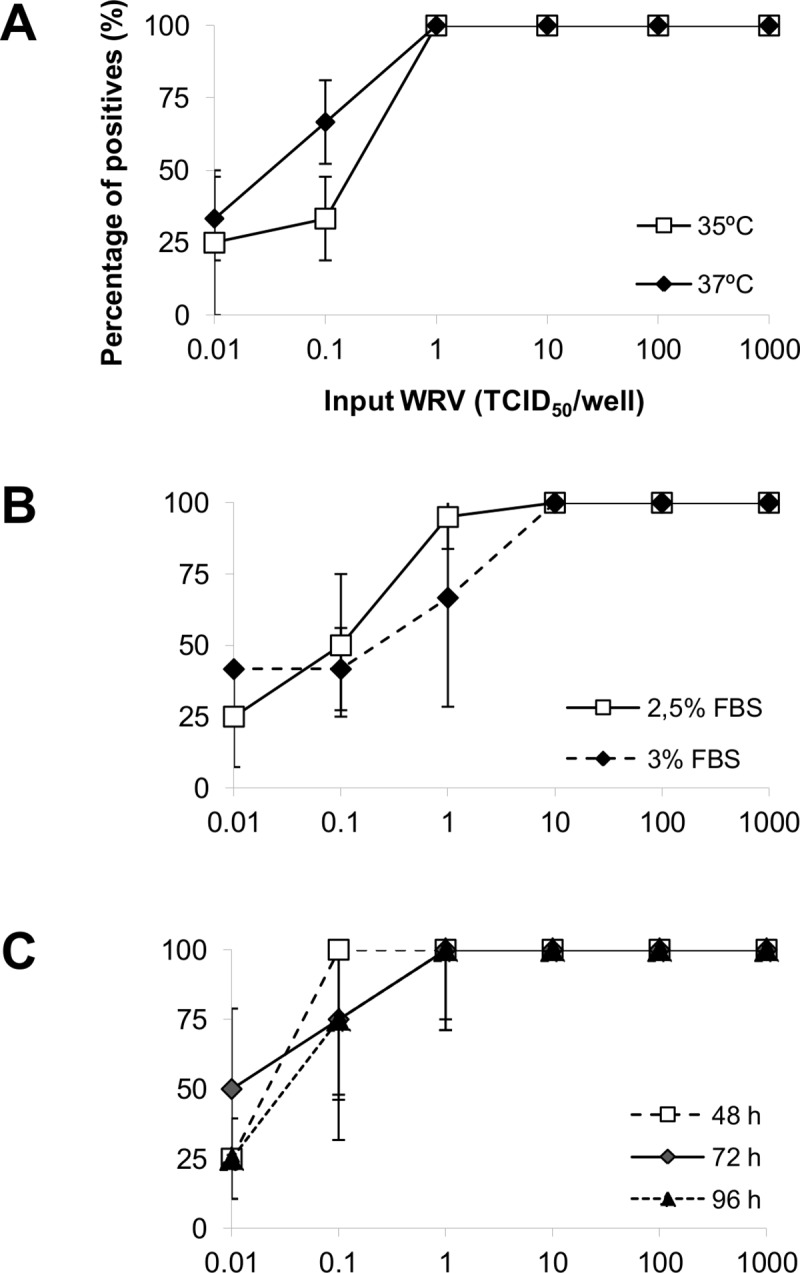
Analysis of optimal culture conditions. Note: BHK-21 cells cultured in 96-well plates at a density of 3.3 x 10^4^ cell/cm^2^ for 24 h were inoculated with 10-times serially diluted WRV (range 0.01–1000 TCDI_50_/well; 4 wells each dose) in culture medium. Cells were incubated (A) for 72 h in DMEM-HAM supplemented with 2.5% fetal bovine serum (FBS) at 35°C or 37°C, (B) for 72 h in DMEM-HAM added 2.5% or 3% FBS at 37°C, or (C) for 48, 72 ou 96 h in DMEM-HAM 2.5% FBS at 37°C. After incubation, cells were fixed and DFA was carried out to identify viral positive cells. TCID_50_, tissue culture infective dose 50%.

To determine the optimal conditions for viral recovery and detection, the effects of culture medium supplementation with FBS, temperature, and period of incubation were analyzed. BHK-21 cells previously cultured in 96-well plates at a density of 3.3 x 10^4^ cells/cm^2^ for 24 hours, were inoculated with serial 10-fold diluted WRV (from 0.01 to 1000 tissue culture infective dose–TCID_50_/well; 4 wells for each dose) in culture medium. Cells were then tested using DFA as described below. Data from 3 independent experiments are shown in Fig 1. Viral production usually occurs using BHK-21 cells in suspension at 33°C, however, the incubation of BHK-21 monolayer cells at 37°C resulted in slightly higher viral recovery. Supplementation of culture medium with FBS (Fig 1B) and incubation period (Fig 1C) did not affect viral recovery rates. Therefore, the cells were cultured in medium supplemented with 2.5% FBS at 37°C for 72 h, in following experiments.

### Evaluation of subculture technique to increase viral recovery

Subculturing techniques may increase viral proliferation and may allow cells to bypass the effect of additives and high load of inactivated virus particles on the infectivity of the residual live viruses. For this experiment, 5 mL of the intermediate product of the vaccine (IVS) was diluted in 15 mL of DMEM-HAM nutrient mixture and 2.5% FBS and spiked with 20 TCID_50_ of WRV. The mixture was inoculated in BHK-21 cells previously cultured in 96-well plates, and incubated at 37°C for 2 h, the used medium was then replaced with fresh culture medium and the plate was further incubated at 37°C for 72 h (original culture). Aliquots of culture supernatant (50 μL) were transferred onto freshly cultured BHK-21 cell plate, culture medium was added and cells were further incubated for 72 h, at 37°C (first subculture). This procedure was repeated one more time after the incubation period (second subculture). Cells in the culture plates were evaluated by the DFA test. Table 1 shows data from 3 independent experiments. The number of positive wells did not increase as the subculture proceeded, hence this technique was not included in the following experiments.

**Table 1:** Effects of subcultures on residual live virus recovery.

	Percentage of positive wells/plate		
Exp. No.	Original culture	First subculture	Second subculture
1	23	7	7
2	26	8	6
3	19	9	2

### Determination of samples used in the *in vitro* assay

Different samples obtained by the production of veterinary inactivated rabies vaccine were tested to determine the best-suited product for analysis. The intermediate products, such as IVS, FIVS containing thimerosal, and HFIVS containing aluminum hydroxide, were diluted in a ratio of 1:4 in DMEM-HAM with 2.5% FBS and inoculated in BHK-21 cells previously cultured in 96-well plates, followed by incubation at 37°C for 2 h. The used medium was replaced with fresh culture medium and the plate was incubated at 37°C for 48 h. As the inoculation of BHK-21 cells with samples containing aluminum hydroxide and thimerosal resulted in cell death, IVS was selected as the sample for *in vitro* assay.

### *In vitro* cell-based DFA and RT-qPCR assay

Based on the results described above, a new *in vitro* assay combining viral recovery through cell culture followed by direct immunofluorescence assay with RABV quantification by RT-qPCR was formulated. For this assay, 5 mL of intermediate product of the rabies vaccine production process, IVS, was diluted in 15 mL of culture medium. 200 μL of the solution was inoculated per well for 2 h at 37°C in BHK-21 cells cultured in a 96-well plate. Used media was replaced with fresh culture medium and the plate was incubated at 37°C for 72 h. The culture medium was removed and cells were fixed to perform DFA. Aliquots were taken from the initial time of incubation and at the end of incubation period, RNA was extracted and RT-qPCR was performed to quantify RABV present in samples.

The duplex RT-qPCR assay for quantification of RABV using β-actin as an internal control was developed by our group and was demonstrated to efficiently quantify RABV present in IVS samples with a limit of quantification of 10^1^ TCID_50_/mL [10]. This assay may be used successfully to evaluate rabies virus inactivation by comparing the quantification results from the time of incubation to the end of the incubation period.

For residual live viruses, results may be considered satisfactory when DFA is negative and RABV quantification by RT-qPCR at 72 h is equal or lower than the quantification at 0 h and unsatisfactory when DFA presents at least one positive well and 72 h RABV quantification is above of quantification limit (10^1^ TCID_50_/mL) and is higher than that observed at 0 h.

### Detection limit of the *in vitro* assay

**Fig 2:**
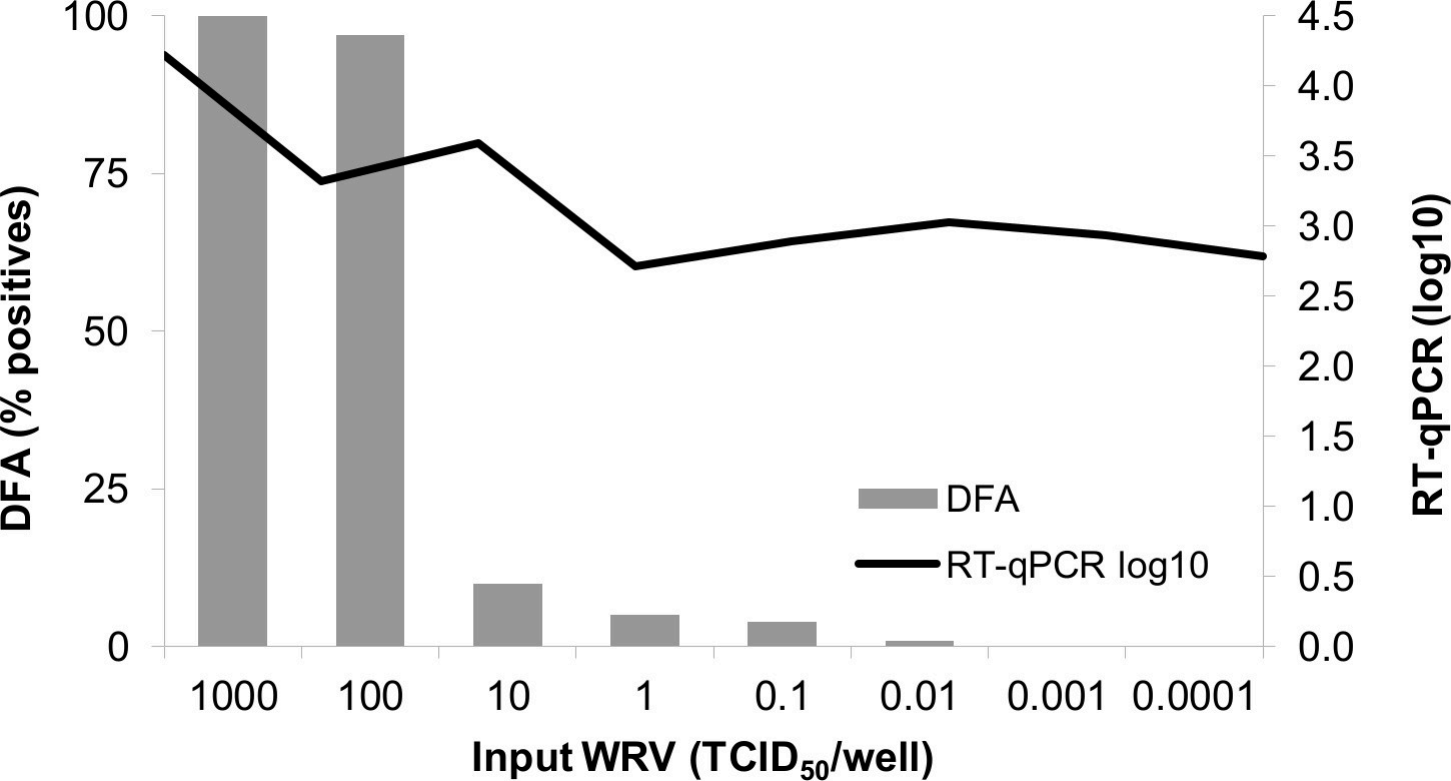
Determination of limit of detection of *in vitro* assay. Note 1: Percentage of positive wells from DFA assay (gray bars) and quantification rates (log10) of RABV at 72 h of incubation with duplex RT-qPCR (black line) calculated as the mean from 3 independent experiments. Note 2. For the sample to be considered positive in the DFA test, it must have at least one positive well, while for RT-qPCR positivity, the quantification obtained in RT-qPCR at 72 h p.i. must be above the quantitation limit and higher than that observed at 0 h”.

The limit of detection (LOD) was initially evaluated by performing the *in vitro* assay with a serial 10-fold diluted WRV (from 10^−4^ to 10^3^ TCID_50_/mL) inoculated in 92 wells with BHK-21 cells (Fig 2). It was determined that the viral recovery assay has the LOD of 10^−2^ TCID_50_/mL, as no positive well was detected bellow this concentration. However, the RT-qPCR assay has a higher LOD, of 10^1^ TCID_50_/mL, considering that lower concentrations resulted in values with less than 0.5 log10 difference.

### Comparison of sensitivity between *in vivo* and *in vitro* assays

**Fig 3:**
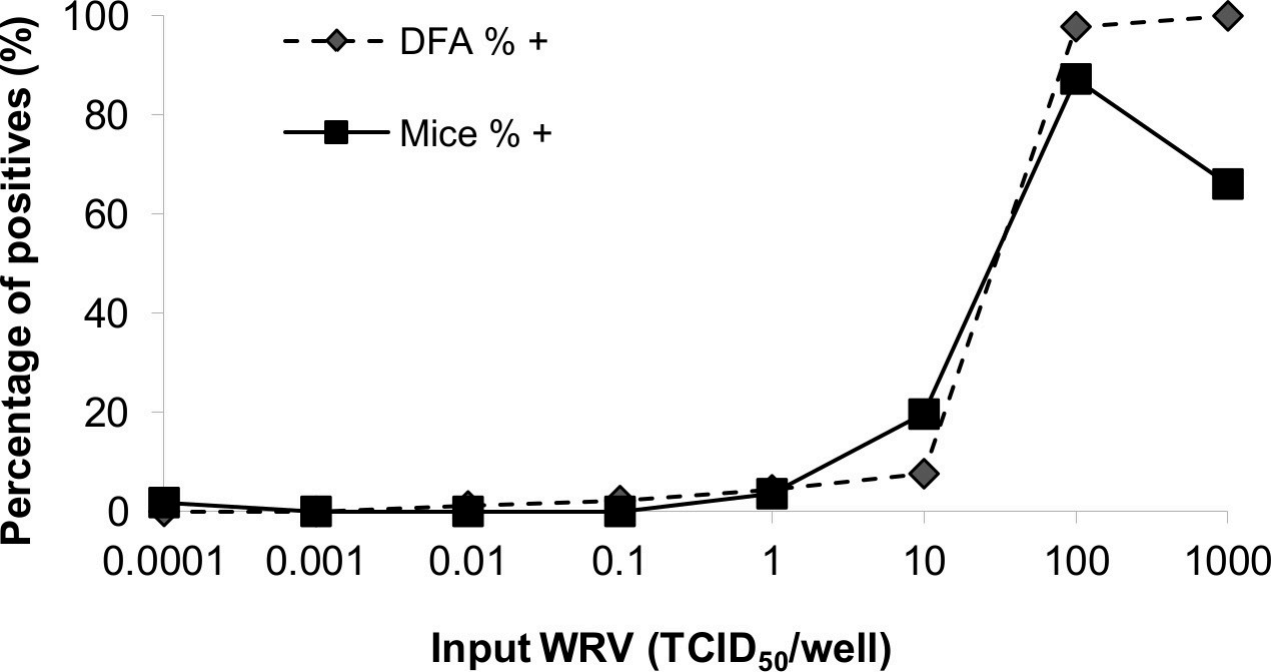
Comparison of sensitivity between *in vivo* and *in vitro* assays. Note: Percentage of positive mice in the *in vivo* assay was calculated as (number of dead mice/56 adult and neonatal mice) x 100 and that of well in the *in vitro* assay was calculated as (number of positive wells/92) x 100. Data is presented as the mean of 3 independent experiments.

The sensitivity of the *in vivo* and *in vitro* assays was compared by analyzing a serial 10-fold diluted WRV (from 10^−4^ to 10^3^ TCID_50_/mL) inoculated in 92 wells with BHK-21 cells and in 56 adult and neonatal mice. Fig 3 shows the mean data of 3 independent experiments. Although the detection percentage was very similar throughout the test, the median lethal dose (LD_50_) values in mice and median cell culture infective dose (CCID_50_) value from the *in vitro* assay calculated using probit analyses, with the NCSS Statistical Software (US), were 67.48 (± 23.20) and 19.70 (± 3.33) TCID_50_/mL, respectively, suggesting that the *in vitro* assay is 3.42 times more sensitive than the *in vivo* assay.

### Initial evaluation of intermediate products of veterinary rabies vaccine production

**Fig 4:**
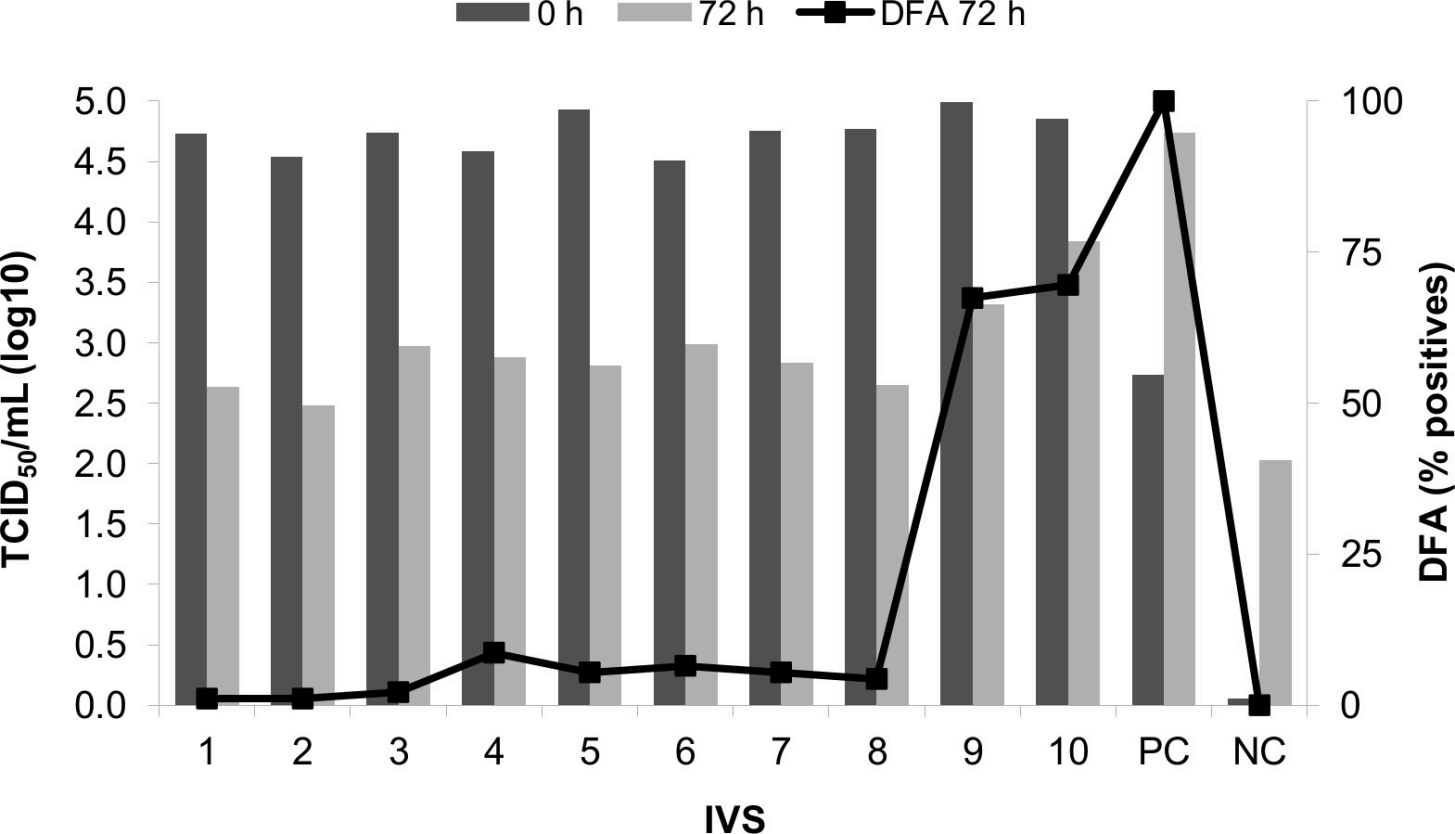
Evaluation of inactivated viral suspension samples. Note: Percentage of positive wells in *in vitro* viral recovery assay (black squares) and quantification rates (log10) of RABV by RT-qPCR at 0 h (dark gray bars) and at 72 h (light gray bars) of incubation. PC, positive control– 2 μL of WRV, 10^5,78^ TCID_50_/mL; NC, negative control–culture medium.

**Fig 5:**
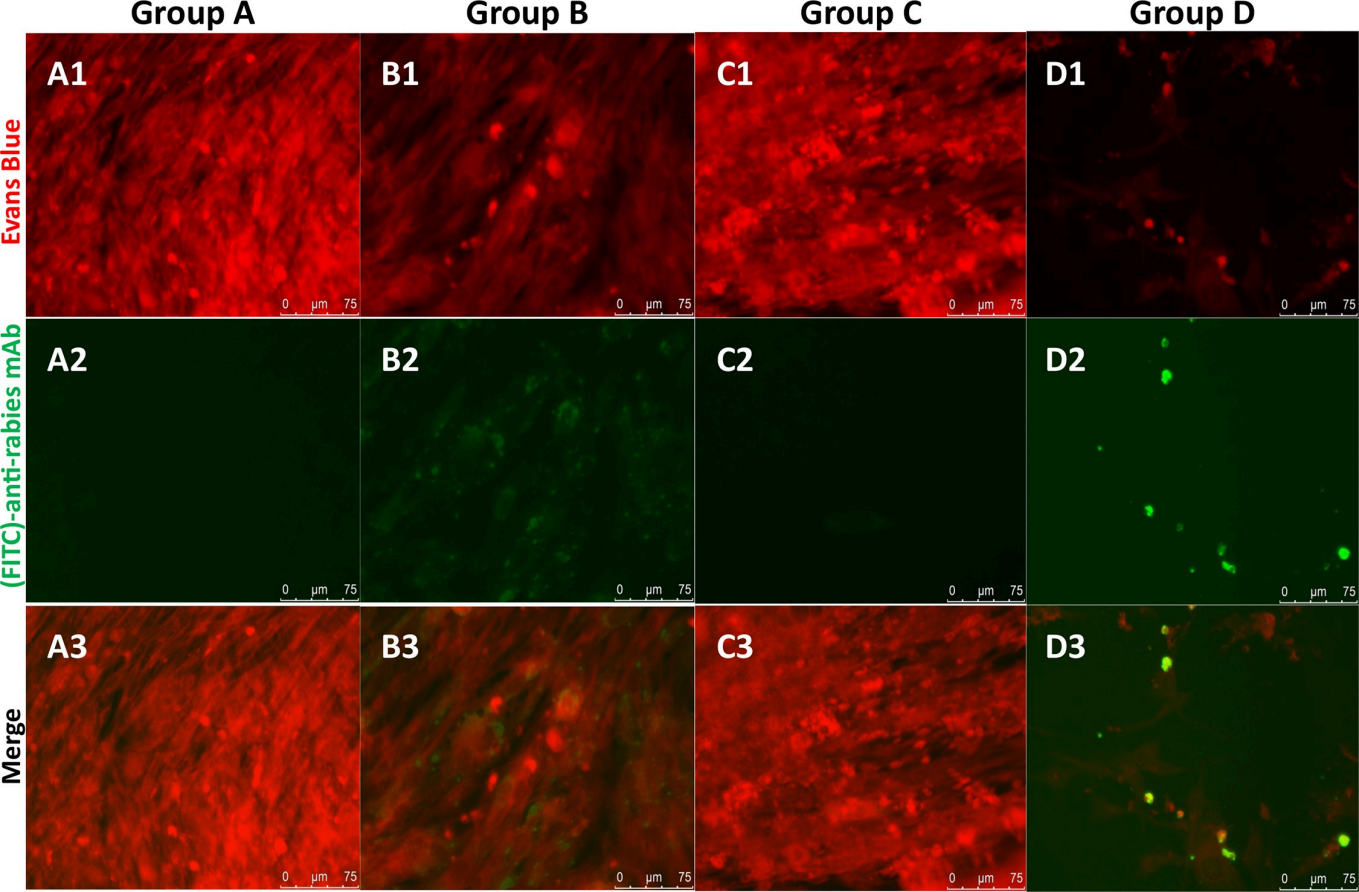
DFA of BHK-21 cells. Note: cells incubated with (Group A) culture medium–negative control, (Group B) 2 μL of WRV, 10^5,78^ TCID_50_/mL–positive control, (Group C) satisfactory IVS sample, and (Group D) unsatisfactory IVS sample, 72 h p.i. Original magnification, 40x.

Once the *in vitro* assay was standardized, we evaluated 10 samples of the intermediate products (IVS) of veterinary rabies vaccine that were satisfactory tested by the *in vivo* residual live virus assay. Fig 4 shows that 8 of the IVS samples presented low to zero positive wells in DFA and low rate of RABV quantification at 72 h, classified as satisfactory by the *in vitro* residual live virus assay. However 2 samples resulted in a high percentage of positive wells in DFA and RABV rates at least 1 log higher than the others, classified as unsatisfactory results. The DFA pattern is shown in Fig 5.

### Confirmation of positive samples

**Fig 6:**
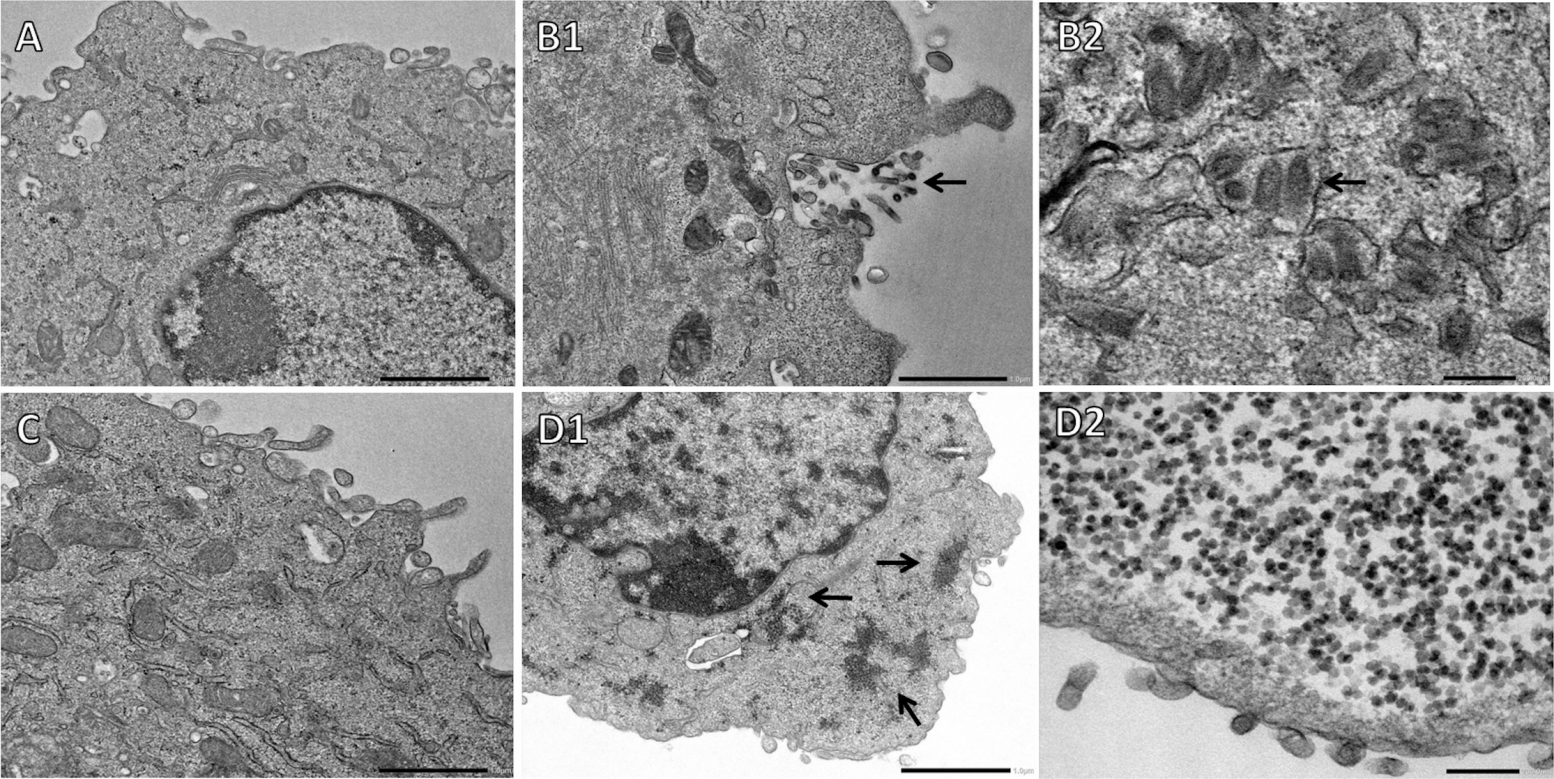
Transmission Electronic Microscopy of BHK-21 cells. Note: cells incubated with (A) DMEM-HAM 2.5% FBS–negative control (1 μm), (B1 and 2) WRV–positive control (1 μm and 200 nm), (C) IVS–(1 μm) and (D1 and 2) IVS+ (1 μm and 200 nm) at 37°C for 72 h. Arrowheads in (B) indicate rabies virus particle, arrowheads in (D) indicate unconfirmed virus particles.

In order to confirm the positive results observed above, the cells inoculated with negative and positive IVS samples were analyzed by transmission electronic microscopy (TEM). As presented in Fig 6, cells inoculated with IVS–showed normal structures (Fig 6C); however, incubation with IVS+ samples resulted in the production of uncharacteristic intracellular viral particles (Fig 6D), which were quite different from the rabies viral particles found in the control samples (Fig 6B).

**Fig 7:**
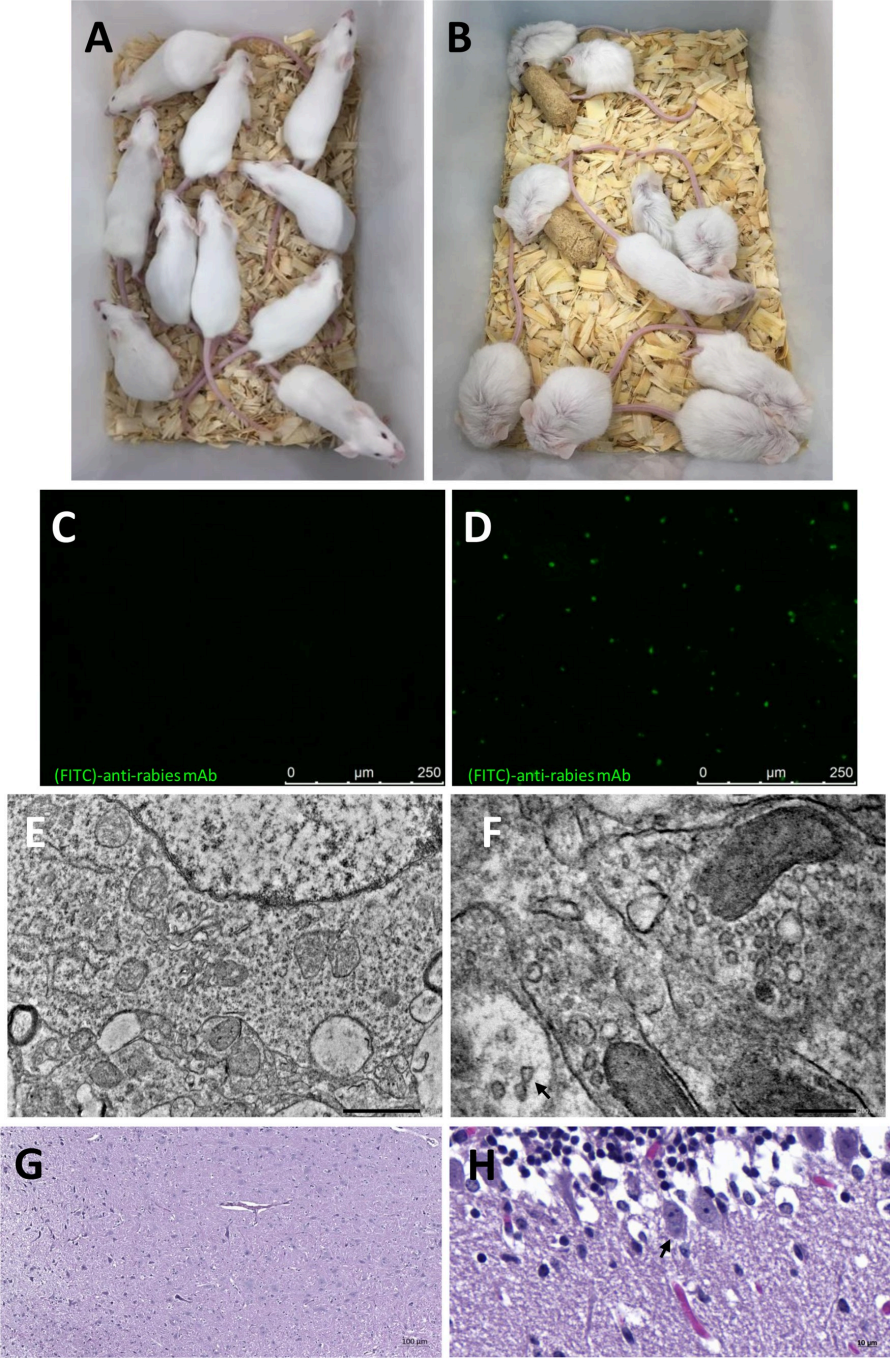
Confirmation of infectivity *in vivo*. Note: (A) mice injected intra-cerebrally with IVS–, 21 days after inoculation showing no clinical signs of rabies; (B) mice injected intra-cerebrally with IVS+, 5 days after inoculation showing specific rabies symptoms, such as ruffled fur, hunched back, slow movements and paralysis; (C) brain DFA of mouse injected intra-cerebrally with IVS–(40x); (D) brain DFA of mouse injected intra-cerebrally with IVS+ (40x); (E) brain TEM of mouse inoculated with IVS–(1 μm); (F) brain TEM of mouse inoculated with IVS+, arrow indicates rabies virus particles (200 nm); (G) brain H&E stain of mouse inoculated with IVS–, basal ganglia with normal cellularity (50x); (H) brain H&E stain of mouse inoculated with IVS+, Purkinje cell with Negri body–arrow (400x).

These findings urged to the need to verify the infection capability of the unidentified viral particles. The infection capability was tested by conducting an *in vivo* residual live virus assay using IVS–and IVS+ samples. Fig 7 shows the results of the confirmatory tests. Fig 7A shows normal healthy mice inoculated with IVS–and Fig 7B shows mice inoculated with IVS+, exhibiting specific clinical signs of rabies. Brain samples from mice with and without rabies symptoms underwent the Confirmatory DFA test was performed on brain samples from mice with and without rabies symptoms (Fig 7D–7C, respectively). Subsequently, samples were analyzed by TEM and H&E staining. Viral particles much like the ones seen in control samples were also seen in IVS+ inoculated brain samples (Fig 7F). The histological analysis resulting from brain samples of mice inoculated with IVS–, with normal looking structures is shown in Fig 7G, while the analysis with IVS+ presenting lesions typical of rabid infection, including presence of Negri bodies, is shown in Fig 7H.

## Discussion

Efforts are being made worldwide to reduce, refine and replace the use of laboratory animals in general science research as well as quality controls assays which are needed to ascertain the quality and efficiency of pharmaceutical products, however, there is still a lot to be done. The quality control procedures for release of rabies vaccine batches and the use of *in vivo* assays are still essential for potency, inactivation, safety and pyrogenicity tests [11,14,15].

Various alternatives to the potency assay have already been developed, for instance incorporation of non-lethal humane endpoints and anesthetics to the NIH test, reducing the number of vaccine dilutions and animals per group, and ultimately replacement of the NIH test by the serological *in vitro* methods such as ELISA [16]. As for the safety test, most international committees question its validity and endorse the discontinuity of the test altogether [7].

For the residual live virus test, regulatory agencies disagree on the recommended assays depending on the use of the vaccine. The WHO recommends a cell-based assay with a duration of 21 days for human rabies vaccines, as does the European Pharmacopeia (EP) [17,18]. However, for the veterinary vaccines, the *in vivo* assay conducted in mice is still allowed by the OIE, the United States Department of Agriculture (USDA) [7], the EP [14] and the Japanese Pharmacopeia (JP) [19], as well as the Brazilian Ministry of Agriculture, Livestock and Supply [11]. Reports of novel *in vitro* assays for the assessment of rabies vaccine inactivation have been published with promising results [19], but further evaluation of their quality assurance and applicability in routine testing is needed.

In this paper we present a new *in vitro* assay for detection of the residual live virus in intermediate products of veterinary rabies vaccine production, combining viral isolation techniques with direct immunofluorescence detection and viral quantification by using molecular methods.

Based on the WHO recommendations, that the test for presence of live virus should be done with the cell line previously used for vaccine production, we used only BHK-21 cells in the development of this assay [17]. This lineage is widely employed for rabies virus isolation in vaccine production, as well as for the diagnosis of the disease, owing to its easy maintenance and high sensitivity to this virus [20–23]. The optimal culture conditions found in this study differ slightly from the previous reports in relation to FBS supplementation [8,19,22–24] and cell concentration [19,22,23,25,26], nonetheless were proven to obtain the best viral recovery rates. Subculture however did not produce satisfactory results, contradicting the previously published study [19], and therefore it was not implemented in the assay. During the standardization of subculture technique, we carried out various methods to increase viral recovery, as trypsinization, longer incubation time, among others. But, we could not increase the virus infectivity level. However, more sensitive assays should be carried out since virus may not be detected by DFA and RT-qPCR in the supernatant if virus infectivity level is extremely low.

There are several ways to quantify rabies virus in cell culture, such as focus forming units (FFU) and TCID50. FFU is a rapid method for virus titration of cell lines that do not form plaques and that also do not exhibit visible cytopathic effects (CPE), it applies the use of labeled antibodies to identify infected cells, thus one identified infected cell equals to 1 FFU. TCID50, on the other hand, measures the titer of virus capable of infecting and causing CPE in 50% of the tissue culture over a period of time. The two measuring units are not comparable, since one cannot determine how many virus particles are needed to infect 50% of the tissue culture. In this assay we combined the two techniques, for the rabies virus does not produces visible CPE, using a fluorescent-labeled antibody to identify the infected cell to calculate the TCID50 titer.

Analysis of the intermediate products of rabies vaccine production process, clearly showed that the preservatives and adjuvants used in its formulation interfere negatively with conduction of the *in vitro* assay. As toxic agents, they prevent cell growth sabotaging the test, effect already showed by Blum et. al., 1998 [27]. We therefore concluded that inactivated viral suspensions with sucrose added as a stabilizer, that configures as the intermediate product of the rabies vaccine production processes, prior to addition of the preservative thimerosal and aluminum hydroxide as an adjuvant, may be used as samples for the residual live virus assay.

The duplex RT-qPCR assay developed earlier by our group proved to be a sensitive method of viral quantification, quantifying live as well as inactivated rabies virus. This assay may be used to quantify rabies viruses at different times of sample incubation in order to determine the rate of viral replication, and as a result, it may help to identify unsatisfactory inactivated samples. The combination of the viral recovery techniques, cell culture and molecular quantification ensures a robust assay, and provides reliable results.

By combining the two techniques we were able to achieve an *in vitro* assay reasonably more sensitive than the *in vivo* test. *In vivo* tests are known to have high variability rates, allows analysis of small amount of samples, and use large quantities of animals, while the *in vitro* assays have the advantage of analysis of significant volumes of samples with considerably less inter-assay variability. The detection limit of the *in vitro* assay (10^−2^ TCID_50_/mL) was notably lesser than the reported limits [19,27], however a clear comparison cannot be made as the measuring units are not interchangeable. Also, different cell and viral lineages have been used by studies, which further complicate the comparison.

After standardization of the *in vitro* assay, we proceeded to its initial validation, where 10 samples of the intermediate vaccine products were tested. Our assay was found to be very sensitive and helped to identify viral suspension samples with residual viable rabies virus particles, which were later confirmed by *in vivo*, TEM, and histological analyses. This strongly supports the need to implement a more sensitive assay in vaccine quality control schemes.

Another limitation to our assay was the nature of sample used in the analysis, as most rabies vaccines contain preservatives and adjuvant agents in their formulation. The *in vitro* assay may be implemented only in the quality control of intermediate products, though further analysis may enable us to perform this assay in the final batches of vaccines that do not carry such chemicals. Furthermore, the use of this assay as an internal quality control could reduce the number of mice used for *in vivo* testing and shorten the quarantine time, as it takes only 4 days for the results of residual live virus to be released once compared to the 21 day assays described by the EP and WHO [14,17].

To conclude, we have developed a new *in vitro* assay for sensitive detection of residual live virus in inactivated veterinary rabies vaccines. This assay helps to identify unsatisfactorily inactivated intermediate products, and hence its implementation in quality control schemes of rabies vaccine production is important. In addition, this method is more practical, inexpensive and less time consuming. Results are produced in just 4 days, which allows expedition of the quality control of vaccines. The *in vitro* assays may help to reduce 2/3^rd^ of the number of laboratory animals being used for this purpose, as it may be implemented in the intermediate quality control of inactivated rabies vaccine production. However, further validation of the technique is still required to verify its applicability in production. And even with the assay’s validation completed, its implementation will only be possible after extensive alterations of officials regulations.
